# Development of protocols for the first serial block-face scanning electron microscopy (SBF SEM) studies of bone tissue

**DOI:** 10.1016/j.bone.2019.115107

**Published:** 2020-02

**Authors:** Patricia Goggin, Elaine M.L. Ho, Helmut Gnaegi, Stuart Searle, Richard O.C. Oreffo, Philipp Schneider

**Affiliations:** a Bioengineering Science Research Group, Faculty of Engineering and Physical Sciences, University of Southampton, Southampton, UK; b Diatome Ltd, Helmstrasse 1, 2560 Nidau, Switzerland; c Gatan UK, Abingdon, OX14 4RY, UK; d Bone and Joint Research Group, Centre for Human Development, Stem Cells and Regeneration, Faculty of Medicine, University of Southampton, Southampton, UK

**Keywords:** 3D imaging, Bone, High resolution, Osteocyte, SBF SEM, Serial block-face scanning electron microscopy

## Abstract

There is an unmet need for a high-resolution three-dimensional (3D) technique to simultaneously image osteocytes and the matrix in which these cells reside. In serial block-face scanning electron microscopy (SBF SEM), an ultramicrotome mounted within the vacuum chamber of a microscope repeatedly sections a resin-embedded block of tissue. Backscattered electron scans of the block face provide a stack of high-resolution two-dimensional images, which can be used to visualise and quantify cells and organelles in 3D. High-resolution 3D images of biological tissues from SBF SEM have been exploited considerably to date in the neuroscience field. However, non-brain samples, in particular hard biological tissues, have appeared more challenging to image by SBF SEM due to the difficulties of sectioning and rendering the samples conductive. We have developed and propose protocols for bone tissue preparation using SBF SEM, for imaging simultaneously soft and hard bone tissue components in 3D. We review the state of the art in high-resolution imaging of osteocytes, provide a historical perspective of SBF SEM, and we present first SBF SEM proof-of-concept studies for murine and human tissue. The application of SBF SEM to hard tissues will facilitate qualitative and quantitative 3D studies of tissue microstructure and ultrastructure in bone development, ageing and pathologies such as osteoporosis and osteoarthritis.

## Highlights

• Introduced SBF SEM for hard tissues, beyond current limitation to soft tissues only.
• Realised concurrent high-resolution 3D imaging of soft & hard bone tissue components.
• Imaged the osteocyte and lacuno-canalicular networks simultaneously using SBF SEM.
• Provided first SBF SEM proof-of-concept studies for murine and human tissue.
• SBF SEM will provide new insights in bone mechanobiology, growth, ageing & pathology.

## Graphical abstract

**Figure:**
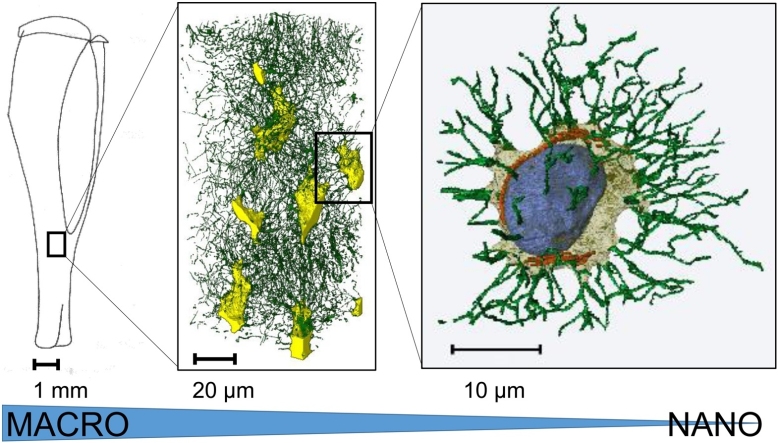

## 1 Introduction

**Fig. 1:**
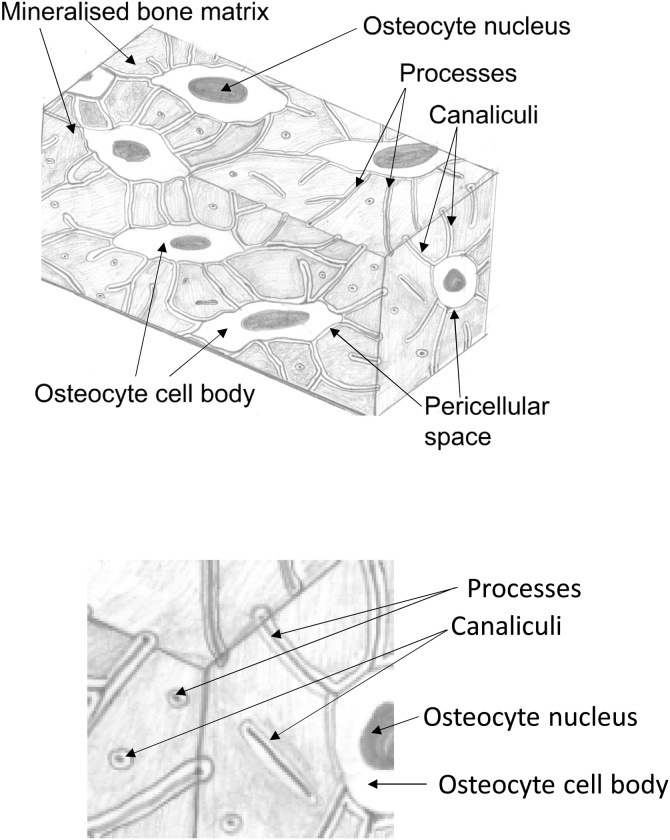
**Schematic views of the osteocyte and lacuno-canalicular networks (ON&LCN)**. The osteocytes and their processes are housed within the mineralised bone matrix in a system formed of (osteocyte) lacunae and interconnecting canaliculi.

Bone is a dynamic tissue which is continually resorbed, formed and remodelled during growth, repair, ageing and disease. An imbalance of formation and resorption is characteristic of bone pathologies such as osteoporosis. Osteocytes, the most abundant bone cells, are typically ovoid cells approximately 15-20 μm long, surrounded by a pericellular matrix [1]. The osteocytes and their processes form the osteocyte network (ON – soft tissue), which is housed within the lacuno-canalicular network (LCN – hard tissue), a system of voids and channels in the calcified bone matrix (Fig. 1). Evidence has accumulated that osteocytes are the ‘orchestrators’ of mechanobiology, responsible for the sensation of mechanical signals and the subsequent transmission of appropriate biochemical signals to osteoblasts (bone formation) and osteoclasts (bone resorption) [2]. However, the mechanisms of osteocyte mechanosensation and mechanotransduction are not fully understood. Theories include the ‘interstitial fluid flow’ hypothesis, in which the attachment of cell processes to the bone matrix by proteoglycan tethering elements and adhesion proteins is significant. You and colleagues suggested that during bone loading, fluid in the pericellular space creates a hydrodynamic drag on the bone matrix, which in turn creates tension on the tethering fibres, resulting in mechanical strain experienced by the osteocyte process membrane [3]. An alternative is the ‘direct strain hypothesis’ in which the osteocyte lacuna is considered to act as a mechanical strain amplifier and that the amplification factor is related to the properties of the surrounding (mineralised) bone matrix [4]. Investigation of these hypotheses requires detailed 3D high-resolution imaging of the microstructure and ultrastructure of the osteocyte and its processes within the bone matrix, providing data for computational models and quantitative analysis [5]. This is more than academic curiosity, the acquisition of high-resolution 3D images, their use in conjunction with *in silico* modelling and the elucidation of mechanisms of bone mechanobiology can, ultimately, lead to improved pathways for diagnosis and treatment of bone diseases such as osteoporosis and osteoarthritis.

To date, several imaging methods have been used in this field. Confocal laser scanning microscopy (CLSM) studies have produced quantitative descriptions of spatial variations in the canalicular density (length per bone volume) [6] and characterised the local mechanical environment of osteocytes and osteoblasts from healthy and osteoporotic bone [7]. Most high-resolution imaging of bone in recent years has been carried out using X-ray micro-computed tomography (μCT). μCT techniques have been widely used for high-resolution 3D imaging of hard tissues (reviewed in [5,8]). For conventional (i.e., absorption-based) μCT, image contrast is generated by the difference in X-ray absorption between the hard bone matrix and the soft and hence weakly X-ray absorbing osteocytes, their processes and the pericellular space within the LCN. Laboratory-based μCT, while capable of imaging relatively large volumes in 3D and being non-destructive, is limited in spatial resolution and images the negative imprint of the osteocytes and not directly the osteocytes themselves. Access to synchrotron light sources (large-scale electron accelerators) is limited and while providing increasingly enhanced spatial resolution beyond the diffraction limit of visible light (< 100 nm [9], 50 nm [10], 16 nm [11]) can restrict the examined sample volume. Electron microscopy (EM), including scanning electron microscopy (SEM) and transmission electron microscopy (TEM), offers high spatial resolutions and can provide ultrastructural images of both the hard and soft components of bone but is inherently a 2D imaging technique. Serial section TEM yields highly comprehensive 3D ultrastructural data over large volumes, notably the reconstruction of the nervous system of *C. Elegans* [12], but is a time-consuming and technically demanding imaging technique. In electron tomography (ET), 250 nm sections are tilted by +/- 70 degrees in a TEM, while a series of images is recorded and reconstructed as a 3D dataset. The resulting images resolve details at extremely high spatial resolutions (< 1 nm in ultra-high voltage TEM) but are restricted to very small volumes. Volume SEM techniques, including serial block-face scanning electron microscopy (SBF SEM), serial focused ion beam scanning electron microscopy (FIB SEM) and array tomography (AT) produce 3D EM data of both the mineralised bone matrix and the enclosed cells. These volume SEM techniques involve sequential slicing (SBF SEM and AT) or milling (FIB SEM) and SEM imaging of a tissue block (SBF SEM and FIB SEM) or the sections removed from the tissue block (AT) [13]. Imaging the block face (SBF SEM and FIB SEM), instead of the sections removed from the block face (AT), produces 2D images that are well aligned, thus reducing the need for subsequent image registration to obtain a properly aligned 3D stack of images. See Table 1 and [5,8] for a summary and description of 3D imaging techniques for the osteocyte and lacuno-canalicular networks (ON&LCN).

**Table 1:** **Techniques for 3D imaging of the osteocyte and lacuno-canalicular networks (ON&LCN)**.

**Technique**	**Destructive**	**Soft tissue contrast**	**Nominal spatial resolution**	**Typical volume**	**Limitations**	**References**
**Confocal laser scanning microscopy**	No	Yes	∼200 nm	0.1 mm^3^	Limited depth	[14] [15] [16]
**X-ray μCT**	No	No	< 1 μm	> 10^9^ μm^3^	High radiation dose	[16] [17]
**Synchrotron radiation-based CT**	No	No	< 50 nm	> 10^9^ μm^3^	High radiation dose, limited access to imaging facilities	[18] [19] [20]
**Serial section TEM**	No	Yes	< 1 nm (*x/y*) and ∼60 nm (*z*)	> 10^3^ μm^3^	Time-consuming, complex sample preparation and image processing	[21]
**Electron tomography**	No	Yes	∼2 nm (*x*,*y*,*z*)	> 10 μm^3^	Missing wedge problem	[22]
**Serial focused ion beam SEM**	Yes	Yes	< 5 nm (*x*/*y*/*z*)	10^3^ μm^3^	Limited field of view, slow, destructive	[23] [24]
**Serial block-face SEM**	Yes	Yes	< 10 nm (*x*/*y*) and < 50 nm (*z*)	10^5^ μm^3^	Complex sample preparation, destructive, non-isotropic voxel size	[5]

Abbreviations: μCT – micro-computed tomography, CT – computed tomography, TEM – transmission electron microscopy, SEM – scanning electron microscopy.

In this work, we first provide a historical perspective on SBF SEM and review recent technology developments for modern SBF SEM. We then describe the development of sample preparation and imaging protocols for SBF SEM of bone, which allows simultaneous imaging of soft and hard bone tissue components, providing combined 3D representations of the ON&LCN. Finally, we present the first SBF SEM proof-of-concept studies of bone tissue conducted by employing the proposed sample preparation and imaging protocols.

### 1.1 Historical perspective on SBF SEM

**Fig. 2:**
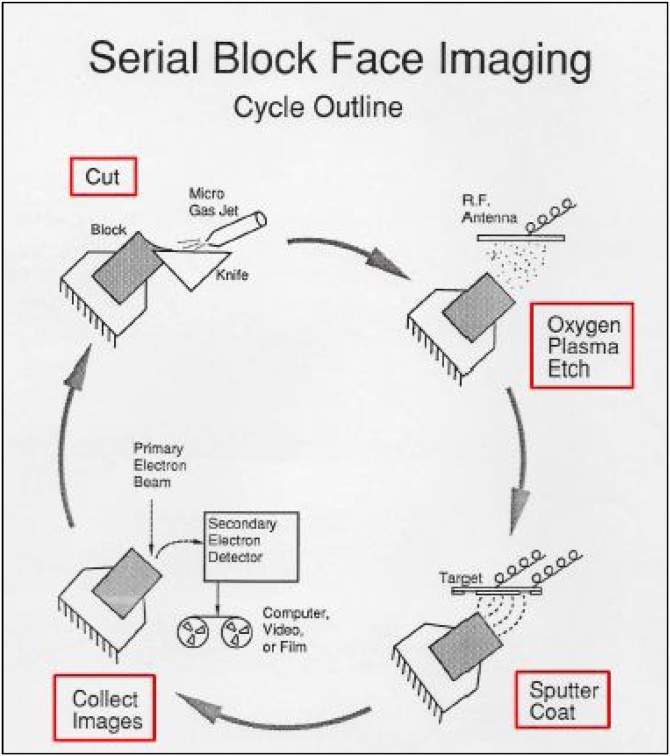
**Cycle of SBF SEM imaging in the prototype apparatus by Leighton and Kuzirian**. In a high-vacuum SEM chamber sections were cut from a resin block using a tungsten-coated glass knife, then the block face was etched with oxygen plasma, improving visualisation of tissue and cellular components. The block face was sputter-coated with gold to render it conductive and allow charge-free imaging before a secondary electron image was recorded and the cycle begun again. The best cycle time for sectioning, etching, coating and imaging was 10 min. Reproduced with the permission of Alan Kuzirian.

Leighton and Kuzirian developed a promising technique for 3D imaging in the 1980s, known as serial block-face imaging (SBFI) [26,27] (Fig. 2). In an SEM chamber sections were cut from a resin block using a tungsten-coated glass knife, then the block face was etched with oxygen plasma, which etches away resin faster than tissue, thus providing relief on the block face and improving visualisation of tissue and cellular components. The block face was sputter-coated with gold to render it conductive and allow imaging that is free of charging artefacts, before a secondary electron image was recorded and the cycle repeated. The quickest cycle time for sectioning, etching, coating and imaging was 10 min. Due to a lack of funding, the contemporaneous development of confocal microscopy, subsequently adopted by many researchers, and due to the limitations of the existing vacuum and imaging technology, such as the restriction to high-vacuum imaging and the collection of data on film or video, SBFI was neglected for a period of time (personal communication, Alan Kuzirian). A meeting between Leighton, Kuzirian and the German physicist Winfried Denk led to further research and development in the early 2000s [28] and the launch of a commercial SBF SEM system through Gatan UK (Abingdon, UK), known as the 3View® system. Alternative platforms such as the Teneo VolumeScope™ SEM (Thermo Fisher Scientific) and Katana Microtome (ConnectomX, Oxford, UK) have also been launched.

### 1.2 Modern SBF SEM

**Fig. 3:**
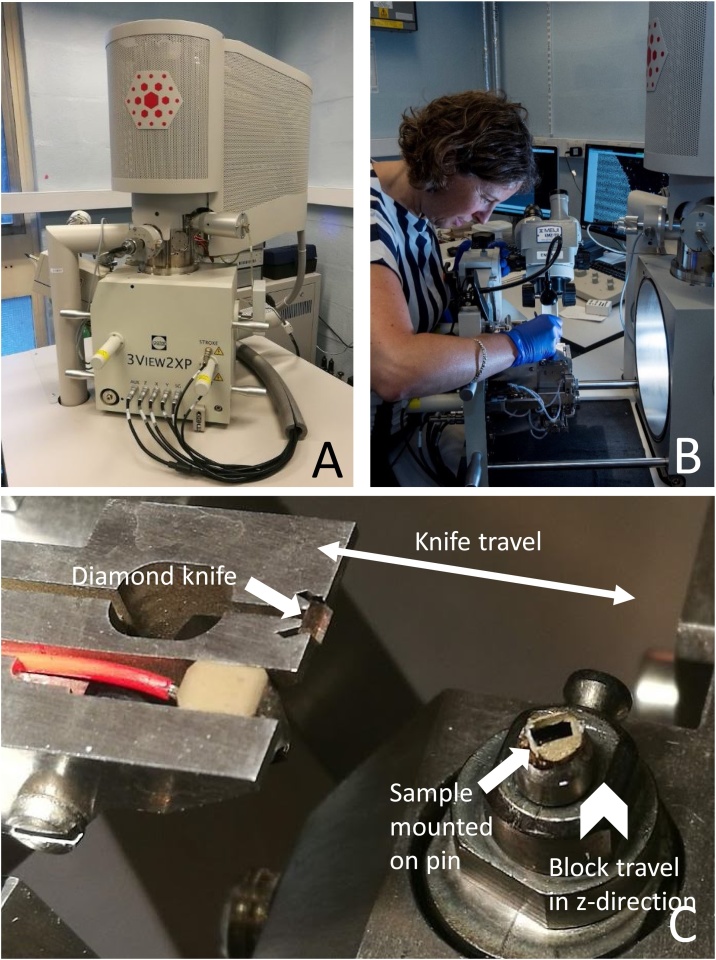
**Modern SBF SEM system**. (A) Gatan 3View® 2XP system fitted in an FEI Quanta 250 field emission gun SEM. The original door supplied with the microscope is replaced by the 3View® system. (B) Loading the block into the 3View® system on the opened microscope door. (C) Detail of sample block mounted on a pin (arrow right) and diamond knife (arrow left) *in situ*. The double-headed arrow shows the travel directions of the knife and the chevron the vertical movement of the block. During operation the diamond knife moves laterally over the sample block (double-headed arrow), which travels upwards for a pre-determined increment (chevron), allowing the removal of a section when the knife returns to its original (‘clear’) position on the left. The block face is imaged while the knife is in this position.

Modern SBF SEM involves automated sequential backscattered electron (BSE) imaging and ultramicrotomy of a sample within an SEM chamber (Fig. 3), enabling subsequent segmentation and 3D reconstruction of cell networks and individual cell ultrastructure, including their quantification by measures such as shape, volume, distribution or connectivity [28]. The advantages of SBF SEM include (i) very high-resolution imaging (typical voxel size ∼10 × 10 × 25 nm^3^) compared to X-ray μCT (typical isotropic voxel size ∼1-10 μm), (ii) a relatively large field of view (up to ∼800 μm^2^) compared to similar techniques such as FIB SEM (∼20 μm^2^), (iii) automated imaging and sectioning, (iv) images that are well aligned to each other due to block-face imaging and (v) sufficient image contrast to visualise and segment the hard and soft tissues of the ON&LCN simultaneously. The disadvantages of SBF SEM include the fact that (i) it is an inherently destructive imaging technique, (ii) the image data generated is typically non-isotropic (different nominal in-plane and out-of-plane resolution), (iii) it involves complex sample preparation protocols and (iv) long imaging times are required. To date, SBF SEM has been widely exploited in the field of the neurosciences [[29], [30], [31]] and more recently, has been applied to various biological specimens, including animal tissues, unicellular organisms and plants, and also to non-biological specimens [32].

The SBF SEM system used in the present study is a 3View® 2XP system, fitted with a 3VBSED detector (Gatan UK, Abingdon, UK) within a FEI Quanta 250 field emission gun SEM (FEGSEM) (FEI, now Thermo Fisher Scientific). The 3View® system can be fitted to FEGSEM instruments including the FEI Quanta 250, 450 and 650, Zeiss Merlin and Sigma and the JEOL JSM-7100 F, 7200 F and 7800 F. The FEI Teneo VolumeScope™ SEM system combines multi-energy deconvolution (optical sectioning using varying accelerating voltages) with mechanical slicing, with the aim of improving the achievable nominal *z*-resolution (slice thickness). The Katana Microtome can be installed on most SEM systems, converting them to an SBF SEM system. The optimum imaging settings may vary depending upon which instrument is used. For example, if low-vacuum mode is not an option, then a lower accelerating voltage is preferable to reduce the build-up of negative surface charges. If a high beam intensity is available, then shorter dwell times, lower accelerating voltages and improved nominal *z*-resolution (thinner slices) will be advantageous.

### 1.3 Application of SBF SEM to hard tissues

CLSM, μCT and EM have provided significant contributions to our understanding of bone structure and mechanobiology. Serial FIB SEM has been used [24,33] to image the ON&LCN covering 2-5 cells at very high spatial resolutions. We perceive that there is a gap for a high-resolution 3D imaging technique that can simultaneously image the soft and hard tissue components of bone over a volume containing a network of up to 100 cells or more, which can be spatially resolved using SBF SEM. SBF SEM has received scant attention to date in the analysis of mineralised tissues in comparison to soft tissues. This is partly due to the availability of the technique. There are roughly 100 X-ray μCT systems in the UK (https://epsrc.ukri.org/files/research/epsrc-x-ray-tomography-roadmap-2018/) compared to currently fewer than 15 SBF SEM systems (personal communication, Paul Spellward, Gatan UK). The lack of uptake to date is also due to the perceived difficulty of ultramicrotomy of harder materials, including the risks of section, block and knife damage, and thus the prevalent research focus on the mineralised hard bone matrix using X-ray methods. We have developed sample preparation and imaging protocols for SBF SEM imaging of bone tissue, and we present here first proof-of-concept animal and human SBF SEM studies.

## 2 Materials and methods

**Fig. 4:**
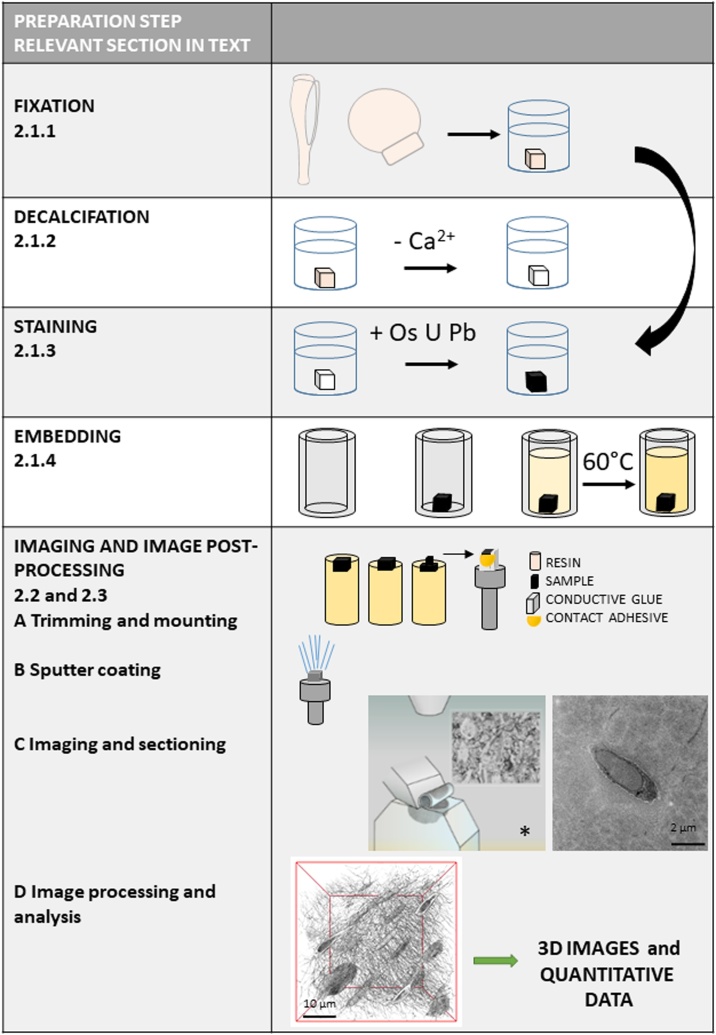
**Workflow diagram for SBF SEM imaging**. Workflow diagram showing the sample processing stages for SBF SEM imaging and subsequent image processing and analysis. The curved arrow shows where decalcification may be omitted. The relevant subsections in the manuscript that cover these individual steps are indicated. * Image with permission from Gatan Inc.

To generate good quality SBF SEM images, the tissue must be rendered electron-dense, producing image contrast and increasing conductivity to prevent charging artefacts which distort the images. The stages of sample processing are similar to those used in preparation for TEM imaging. We will consider the optimisation of each sample processing stage in turn and subsequently the SEM imaging conditions (Fig. 4). We have used murine and human bone samples in these optimisation studies. Section 5 ‘Protocol for SBF SEM imaging of bone tissue’ contains the protocol we considered to be optimal for SBF SEM sample preparation and imaging of bone tissue, with a particular focus on osteocytes with their processes and the surrounding mineralised bone matrix, which forms the osteocyte lacunae and canaliculi (Fig. 1).

### 2.1 Sample preparation

TEM processing involves *Fixation* to preserve the components of the cell ultrastructure, *Staining* with heavy metals (usually osmium and uranium) to increase electron density, *Dehydration* and *Infiltration* with resin followed by *Embedding* and *Polymerisation* to create a block of uniform hardness. Subsequent on-section *Staining* with heavy metal (usually lead) further enhances image contrast. As bone tissue is mineralised to a certain extent, a *Decalcification* step may be included to render the tissue suitable for microtomy. These steps are optimised to (i) preserve the native shape and organisation of the cells, (ii) render the tissue able to withstand the electron beam and the vacuum environment without shrinking or tearing, (iii) provide image contrast between the tissue structures and (iv) make the block of sufficient and uniform hardness to be sectioned easily [35]. It is important to recognise however that each of these steps takes the tissue away from its hydrated *in vivo* condition and this must be considered when interpreting the resulting images.

The principles of TEM specimen preparation apply equally to SBF SEM with the added demands that the tissue should be as conductive as possible in order to allow scanning of the block face without build-up of surface charge. The tissue should also exhibit enhanced electron density to allow for a strong high-contrast BSE signal to be delivered. This in turn facilitates image visualisation, segmentation and quantification. We have optimised each sample preparation step in murine and human tissue and recommend approaches for ON&LCN imaging.

#### 2.1.1 Fixation

Observation of bone structures at a cellular level and in a close-to-native state is challenging since most imaging techniques require tissue processing and/or sectioning, which entail a plethora of chemical and physical changes of the bone tissue [35,36]. Several options exist for EM preparation, preserving the mineral and organic components of the bone matrix and the bone cell structures to various degrees. Chemical fixation typically uses aldehydes, which form crosslinks (covalent bonds) between tissue proteins, arresting the movement of proteins and adding rigidity to the tissue. Perfusion fixation is ideal, given the cells are fixed immediately at the moment of death. However, for ethical and practical reasons, as the entire organism must be sacrificed and large volumes of fixative are required (for example when samples of mammalian origin are used), immersion fixation may be used as an alternative. To this end, tissue should be fixed immediately after excision, not allowed to dry out, thus avoiding unwanted cell collapse. The tissue should be dissected into blocks (< 2 × 2 × 2 mm^3^) to ensure complete penetration of all processing solutions, while being submerged in fixative solution. Mineralised tissue is a challenging material for precise and immediate dissection into such small blocks due to its hardness. Fine-toothed and diamond saws, sharp single-edged razor blades and bone trephines may be used to facilitate production of suitably sized tissue blocks. The most commonly used EM fixatives are glutaraldehyde (C_5_H_8_O_2_) (1-4%) and formaldehyde (CH_2_O) (2-4%). Formaldehyde penetrates tissue quickly, and although the rate of diffusion of glutaraldehyde is slower, glutaraldehyde forms stronger links within biological tissues. The two are often used in combination. Additions to aldehyde fixatives have been shown to improve aspects of bone cell preservation. Acrolein is a small molecule which has been used for its fast penetration qualities [37] and Ruthenium III hexamine trichloride improves fixation of proteoglycans [38]. Fixation should result in images which reflect the native state of living cells, without tissue preparation artefacts such as shrinkage. Fixatives for EM are delivered in a buffered solution. It is recommended that buffers containing phosphate are avoided when fixing bone tissue, as this can lead to the formation of calcium phosphate crystals around and within the cells [39]. Suitable alternatives are 1,4-piperazinediethane sulfonic acid (PIPES) (C_8_H_18_N_2_O_6_S_2_) and sodium cacodylate (C_2_H_6_AsNaO_2_) buffers.

**Fig. 5:**
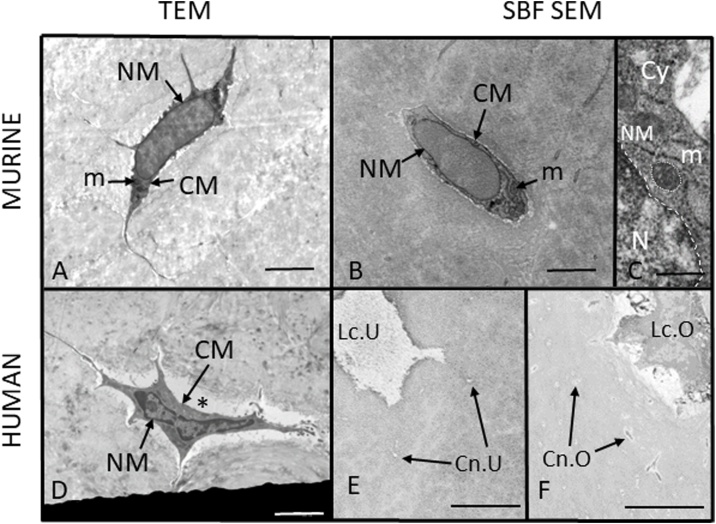
**Assessment of bone sample preparation quality for EM imaging and lacunar occupancy**. (A) TEM image of a murine osteocyte in perfusion-fixed, decalcified bone showing sufficient spatial resolution and image contrast for visualisation of cell ultrastructure. The cell membrane (CM) and nuclear membrane (NM) are intact and regular, the mitochondria (m) show neither swelling nor shrinkage, and there is no cell shrinkage evident. (B) SBF SEM image of a murine osteocyte in perfusion-fixed, decalcified bone showing sufficient spatial resolution and image contrast for visualisation of cell ultrastructure. The cell membrane (CM) and nuclear membrane (NM) are intact and regular, the mitochondria (m) show neither swelling nor shrinkage, and there is no cell shrinkage evident. (C) Detail of cell nucleus (N), nuclear membrane (NM, dashed outline), cytoplasm (Cy) and mitochondria (m, dotted outline) from an SBF SEM image of a murine osteocyte. (D) TEM image of a human osteocyte in immersion-fixed, decalcified tissue showing intact cell membrane (CM) and nuclear membrane (NM). The pericellular space (*) is enlarged, probably due to cell shrinkage. (E, F) SBF SEM images of immersion-fixed, decalcified human bone tissue showing unoccupied (Lc.U) and occupied (Lc.O) osteocyte lacunae and unoccupied (Cn.U) and occupied (Cn.O) osteocyte canaliculi. Scale bars A, B and D = 2 μm, C = 200 nm, E and F = 5 μm.

**Fig. 6:**
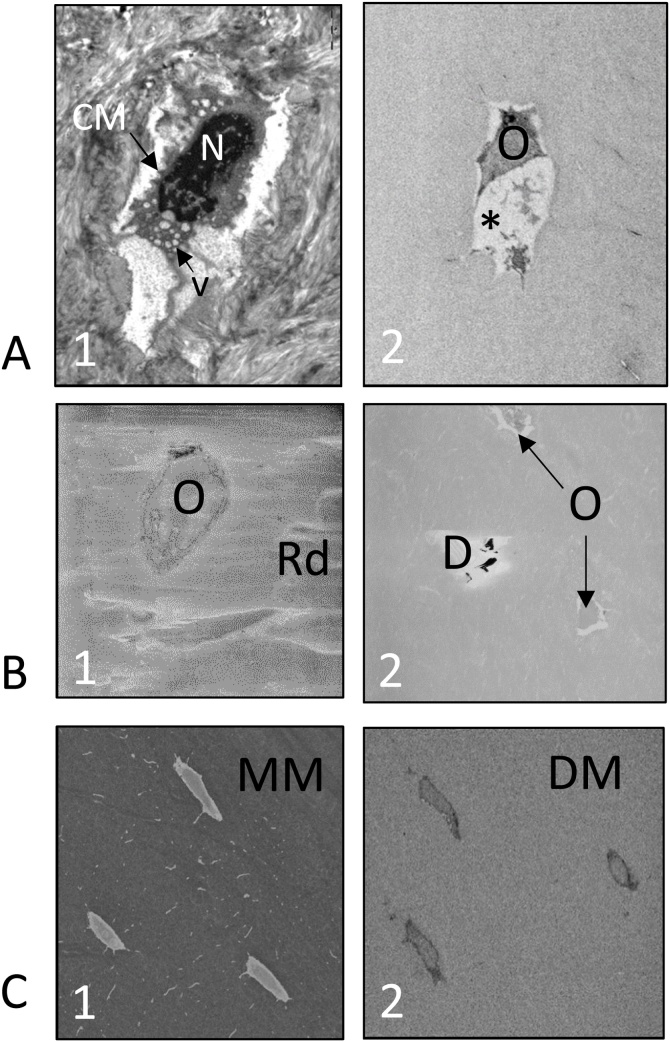
**The effects of sample preparation for EM imaging**. (A) Preservation of cell ultrastructure: (A1) TEM image of a murine osteocyte showing poor preservation of cell ultrastructure. The cell membrane is distorted, the cytoplasm contains vesicles (v) and the nuclear material (N) is clumped; (A2) SBF SEM image of a human osteocyte showing poor preservation of cell ultrastructure and a large shrinkage artefact (*). (B) Problems caused by resin: (B1) TAAB resin-embedded murine bone tissue showing an osteocyte (O) and surface resin damage (Rd); (B2) ALV resin-embedded murine bone tissue showing osteocytes (O) and debris (D) on the surface. Both images, B1 and B2, exhibit reduced contrast. (C) SBF SEM images of decalcified and undecalcified bone tissue: (C1) Undecalcified murine bone tissue, showing osteocytes within the mineralised matrix (MM); (C2) Decalcified murine bone tissue, showing osteocytes within the decalcified matrix (DM).

To demonstrate that SBF SEM provides images at spatial resolutions sufficient to visualise cell ultrastructure, we have compared TEM and SBF SEM imaging of osteocytes. Fig. 5 shows SBF SEM and TEM images of murine and human osteocytes in decalcified tissue. The images exhibit sufficient spatial resolution and image contrast for visualisation of cell ultrastructure and, critically, illustrate the features of cells that are well fixed. The cell membrane (CM) and nuclear membrane (NM) remain intact and regular, the mitochondria (m) show neither swelling nor shrinkage, and there is no cell shrinkage evident. Using SBF SEM, it is also possible to determine whether lacunae are occupied by an osteocyte and similarly, whether canaliculi house a cell process or not (Fig. 5). Examples of poor fixation and preparation artefacts are presented in Fig. 6.

#### 2.1.2 Decalcification

Undecalcified bone tissue can be sawn into wafers, then treated with abrasive to form ground sections (∼10 μm in thickness), with or without resin embedding [40]. Laser ablation microtomy can also be used to cut sections of bone tissue suitable for light microscopy [41]. FIB milling [42,43], cryo FIB milling [43] and Argon ion beam thinning [44] can be employed to generate thin sections for TEM imaging by using a focussed ion beam to erode the specimen to a thin layer. Sections can also be produced using a heavy-duty microtome with a tungsten carbide blade or a diamond knife. However, this can cause tissue damage, such as scratching, section splitting or loss of sample material by pulling the mineralised material across the surface of the block. Knife damage is also possible (see Section 2.2.1 ‘Microtomy’). For smooth, damage-free microtomy the tissue should be of similar hardness to the surrounding resin. Given that epoxy resins are softer than bone, the bone is decalcified to render the tissue mechanically compatible with the embedding medium and the sectioning method.

Decalcification (demineralisation) of bone tissue before processing for EM imaging minimises knife damage and facilitates production of high-quality thin sections. The decalcification process should remove minerals from the tissue without changing the cell structure or introducing other artefacts, and decalcification should render the tissue as easy to cut as non-mineralised tissue without causing damage to either the knife edge or the block face. During decalcification, strong mineral acids (nitric, hydrochloric), weak organic acids (formic, picric) or chelating agents (ethylenediaminetetraacetic acid (EDTA)) remove Ca^+^ ions to make the tissue flexible and easy to section [45]. Strong acids decalcify rapidly, but can affect the stainability of tissue [[46], [47], [48]]. EDTA affects only the bone matrix mineral and thus, has less potential to cause cellular damage, important in EM imaging, but it decalcifies more slowly. The decalcification process can take days or weeks depending on the tissue block size, concentration of decalcifying agent, agitation and temperature [40,49]. Decalcification of bone tissue, which has been embedded in resin, has also been suggested as a method but has produced variable results [39,50,51]. Microwaves can be used to accelerate the decalcification process in bone and teeth and it is also suggested that this can result in a more uniform staining of the tissue [46,52], yet this process is not widely employed. It has been shown using atomic force microscopy (AFM) that decalcification does not affect the integrity of the LCN [53].

We have compared SBF SEM imaging of mineralised bone to bone tissue that has been decalcified using 7% EDTA (Fisher Scientific, Loughborough, UK) for one week, changing the EDTA solution daily (Fig. 6). The advantages of decalcifying bone tissue for SBF SEM include (i) greater ultrastructural image contrast, making automated segmentation of subcellular structures easier, (ii) improved image quality (Fig. 6) and (iii) less damage to the diamond knife (and an extended life time) which will be discussed later in Section 2.2.1 ‘Microtomy’. The disadvantages of decalcification include that (i) the tissue is further from the native state, (ii) the image contrast between the cells and surrounding matrix is reduced, making automatic segmentation of the lacunae more difficult and that (iii) the time required to prepare the decalcified sample is considerably longer than the mineralised sample.

#### 2.1.3 Staining

Contrast in EM imaging depends on the interaction of the electron beam with structures of differing electron density. Soft tissue structures do not have a high inherent electron density, so stains must be attached to the organic molecules to increase image contrast. The effectiveness of the stain is related to its atomic weight, thus the most widely used are heavy metals. Staining can be carried out *en bloc* during sample preparation and/or on grid-mounted sections before TEM imaging, however for SBF SEM, all stains must be in the block before imaging. In addition to enhancing image contrast, heavy metals make tissue more conductive, which reduces charging, reduces the breakdown of resin and thus improves sectioning and image quality.

Osmium tetroxide has long been used as a TEM fixative and tissue stain [54]. While sufficient to provide contrast in TEM imaging, tissue stained with osmium tetroxide alone does not impart enough contrast in SBF SEM imaging where low (2-5 kV) accelerating voltages and a BSE detector are used. Thus, protocols have been developed to increase the impregnation of heavy metals, including the osmium-thiocarbohydrazide-osmium (OTO) method, where thiocarbohydrazide acts as a bridging reagent allowing more osmium to bind to the tissue [55,56], and the ferrocyanide-reduced osmium tetroxide methods (R-OTO) [57,58]. More recent protocols combine these methods with prolonged uranyl acetate and *en bloc* lead aspartate staining [59], tannic acid treatment [60] and uranyl acetate, lead aspartate, copper sulphate and lead citrate [25] to increase the yield of BSEs during imaging. Protocols for staining volumes as large as a whole mouse brain (∼500 mm^3^) have been developed [61,62]. Stains originally developed for TEM imaging, for example zinc iodide, are being trialled and found to be effective as SBF SEM stains. This potentially allows imaging of precious archive material prepared many years in the past, where these stains have been employed [63,64]. Our optimal protocol is based on the ‘Ellisman’ protocol [59], which was originally developed to enhance the contrast of cell membranes in SBF SEM imaging. This protocol has been widely adapted and has gained acceptance in the 3D EM community as an effective staining method.

#### 2.1.4 Embedding

Biological samples for both FIB SEM and SBF SEM need to be embedded in resin which supports the tissue, creates uniform hardness across tissue and resin, allows the block to remain stable, resist shrinkage and maintain integrity in the electron beam. Topographical analysis of radiation damage using TEM and AFM have shown that HardPlus 812 resin and a mixture of Durcupan and Epon resins are suitable for FIB SEM imaging, maintaining stability and dimensional integrity during imaging and processing [65]. To the best knowledge of the authors, no comprehensive comparison of resins for SBF SEM imaging has been carried out to date. At present, all commercially available resins for EM are non-conductive. However, recent developments have suggested that the addition of materials, such as carbon nanotubes or carbon black filler, can produce a conductive resin which reduces charging artefacts and improves spatial resolution [66,67]. It should be noted that while well-dispersed carbon nanotubes or nanoparticles are not problematic, when agglomerated, these additives can cause damage to diamond knives. We have generated and compared SBF SEM data from bone tissue embedded in three resins, namely (i) Agar low viscosity (ALV) resin (a replacement for carcinogenic Spurr resin) (Agar Scientific, Stansted, UK), (ii) Durcupan resin (TAAB Laboratories Equipment Ltd, Aldermaston, UK) and (iii) TAAB resin (TAAB Laboratories Equipment Ltd, Aldermaston, UK) to identify the most suitable for SBF SEM imaging of the ON&LCN. Both Durcupan and TAAB resins showed surface damage at a lower electron dose than ALV resin. Spurr resin, which ALV replaces, has been used previously for the preparation of mineralised bone for light microscopy [68], and our studies here indicate that ALV resin, which has a low viscosity, is easy to use and provides rapid and complete tissue infiltration making it a suitable choice (Fig. 6).

Semi-thin (0.5-1 μm) and/or ultrathin (∼90 nm) sections may be taken and examined under a light microscope and/or using TEM before proceeding to SBF SEM imaging to confirm sample orientation, allow selection of an area of interest and confirm tissue fixation quality. The block face is trimmed with a single-edged razor blade, glass knife or diamond trimming knife to a surface area < 800 μm^2^, with the region of interest (ROI) near the centre and removed from the resin block using a sharp single-edged razor blade. A piece of laboratory film, placed over the surface during removal, can protect against loss of the sample sub-volume. Conductive glue (CircuitWorks Conductive Epoxy CW2400; ITW Chemtronics, GA, USA) is used to attach the sub-volume block to an aluminium pin, which enhances conductivity and thus reduces the build-up of negative surface charge and its adverse effects, such as blurring and distortion of the EM image. The addition of a small amount of contact adhesive (Pattex; Henkel Düsseldorf, Germany) to the edge of the block cut first by the diamond knife (Fig. 4), causes the slices of resin-embedded tissue to adhere to the knife edge. This reduces the risk of free-floating resin sections, which can mask details of the block, obscure apertures or contaminate the detector. Mounted samples are sputter-coated with a layer of metal (Au, Pt or Pd) to further reduce charging (Fig. 4).

### 2.2 SBF SEM imaging

#### 2.2.1 Microtomy

The diamond knife, invented by Morán in 1955 (patent no. US3190047A) [69], was made of the hardest known material at the time of development and is commonly used to produce ultrathin sections (∼100 nm) of tissue for examination by TEM. Diamond knives are sharp and long-lasting, but also delicate, expensive and costly to re-sharpen. The optimum combination of knife angle, sharpness, oscillation and cutting speed is essential for minimising damage to the block face and consequently, avoiding artefacts in the images [70]. The knife can introduce artefacts on both the section removed from the block and on the block face, including but not limited to chatter (an artefact of vibration evidenced by regular stripes perpendicular to the direction of cutting on the block and section), tissue compression/shearing, knife marks on the block face and the removal of hard particles embedded in the tissue. Damage caused by sectioning may not always be visible because SBF SEM images the block face rather than the removed sections, and because it uses BSEs, which originate from deeper in the interaction volume and are less affected by surface topography.

We tested a 3View® knife (Diatome Ltd, Nidau, Switzerland) on mineralised bone tissue prepared as described in our protocol (see Section 5 ‘Protocol for SBF SEM imaging of bone tissue’). After cutting 4000 sections, no evidence of damage was visible on the block face nor the knife edge. The knife edge was subsequently assessed by sectioning a blank epoxy resin block and examining the block face using incident light with a Nomarski differential interference contrast microscope. This technique shows up even the smallest imperfections in the block face as ‘tramlines’. Areas of our knife showed more wear after use on mineralised bone than on decalcified bone, but still enabled good quality cutting and imaging. The knife in current use has taken approximately 10,000 sections and shows no artefacts on SBF SEM images.

#### 2.2.2 Image acquisition conditions

During SBF SEM imaging, the operator must find a balance between image quality, data volume, acquisition speed and sample damage. Table 2 shows how varying acquisition settings can affect image quality. In SBF SEM the BSE detector captures sequential images of the block face controlled by specialised custom software with minimal user interaction. Automatic focus and stigmator checks are used to maintain image quality on long runs (up to 4 weeks; personal communication, Lucy Collinson, Francis Crick Institute, London). Image quality has a large impact on subsequent image segmentation and quantification and hence, must be carefully optimised for each sample type, magnification setting and desired spatial resolution. Suboptimal image data quality can impede or preclude automatic or semi-automatic image segmentation.

**Table 2:** **The effects of changing SBF SEM imaging conditions.** The table shows a summary of SBF SEM imaging conditions and their effects on the output images. Green cells indicate desirable outcomes.

SNR = signal-to-noise ratio.

Working distance, the distance from the point of focus on the sample surface to the final pole piece of the SEM lens, is usually a consideration in SEM imaging, with shorter working distances giving improved spatial resolution but reduced focal depth. Focal depth is not a concern for SBF SEM since a flat surface is imaged. For the 3View®/FEI Quanta system used here the working distance was 6.5 mm, which the operator cannot change.

**Fig. 7:**
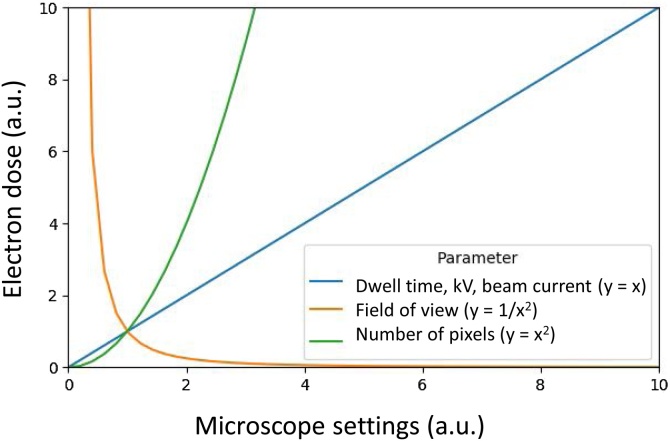
**Empirical relationships between SBF SEM imaging conditions and electron dose**. Varying the operating conditions has an impact on the BSE signal/image quality and the electron dose. Dose is a function of volume (pixel size and slice thickness), energy (accelerating voltage) and beam current (controlled by spot size). Compromises must be made to achieve optimal imaging settings for maximised image quality and optimum cutting quality. kV = accelerating voltage.

The electron dose, the number of incident beam electrons hitting the surface per unit area, is affected by experimental settings including dwell time, beam current and pixel size (Fig. 7). The electron dose can be calculated using the following equation [71]:

(1) $$\text{Electron dose}\;\;\left[{\text{ⅇ}}^{-}/{\text{nm}}^{2}\right]=\frac{\text{Beam current }\left[\text{A}\right]\times 6.24151\text{x}10^{18}\;\left[{\text{ⅇ}}^{-}/\text{C}\right]\times \text{pixel dwell time [s]}}{{\left(\text{pixel size }\left[\text{nm}\right]\right)}^{2}}$$

**Fig. 8:**
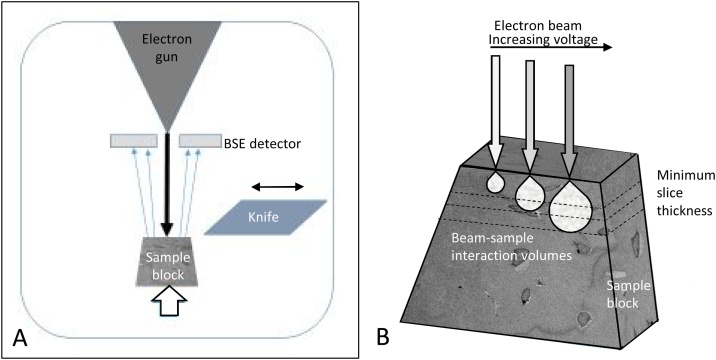
**Beam/sample interactions during SBF SEM imaging**. (A) The incident electron beam (black arrow) interacts with the sample and BSEs (blue arrows) are detected. The block moves upwards (white arrow) and the knife moves horizontally (double-headed arrow) to remove a slice of tissue before the cycle restarts. (B) Increasing accelerating voltages (grey arrows) lead to a greater depth of electron penetration and associated tissue damage. Slice thickness (dotted lines) should be greater than the penetration depth of the beam in order to remove resin which has been affected by the beam and thus, to avoid surface damage in the subsequent image. (For interpretation of the references to colour in this figure legend, the reader is referred to the web version of this article.)

An increased electron dose results in improved image quality (higher signal-to-noise ratio (SNR)), but can cause breakdown of the resin and charging above a certain threshold, leading to image distortion and non-uniform cutting thickness (Fig. 6). Slice thickness should be greater than the electron beam penetration depth (Fig. 8) to ensure that the newly exposed block face is undamaged, important for an artefact-free EM image. We have created an interactive dose calculator to predict best settings and assess the effects of changing experimental conditions (Supplementary 1). The dose calculator applies Eq. (1) and the relationships plotted in Fig. 7 to determine the dose for different experimental settings. The user inputs imaging and cutting settings so that dose and other metrics can be calculated. Other dose metrics, such as energy at a given depth, will also have a bearing on the cutting performance. The dose calculator provides detailed calculations which are of use when predicting imaging conditions.

The maximum dose providing artefact-free images varies with tissue type, sample preparation, slice thickness and experimental (image) settings. Kubota has suggested that a maximum of 20 electrons/nm^2^ is the limit for successful SBF SEM imaging of brain tissue embedded in Durcupan resin [71]. It is a useful exercise to establish the maximum dose for samples prepared by a particular protocol to guide image optimisation. The maximum dose can be established by starting with recommended settings by the manufacturer (for the 3View®/FEI Quanta 250 system: accelerating voltage = 2 kV, spot size = 3, image size = 1k × 1k, pixel size = 15 nm, dwell time = 4 μs and slice thickness = 50 nm) and visually assessing caused tissue damage using both the secondary electron detector and BSE detector. If tissue damage is observed, the dwell time should be reduced and imaging restarted until no tissue damage can be visually detected anymore. Using the dose calculator (Supplementary 1), we determined that the maximum artefact-free dose for our samples and imaging setup was 15.6 e^−^/nm^2^ at 50 nm slice thickness, which is consistent with the suggestion of Kubota [71]. The tolerable electron dose will vary with tissue staining, resin hardness, knife sharpness and focusing of the electron beam.

#### 2.2.3 Optimisation of SBF SEM imaging conditions

We carried out a series of image analyses to justify our choice of SBF SEM settings. Ideally, SBF SEM would produce high-resolution, noise-free images, where features of interest can be segmented easily, in an automatic fashion. Noise is unavoidable, but the effect is minimised by careful setting of the experimental conditions (Table 2). We compared SNR, contrast-to-noise ratio (CNR) and sharpness across a range of accelerating voltages, spot sizes, vacuum levels and slice thicknesses, using both decalcified and mineralised murine tissue. SNR and CNR are common non-dimensional figures of merit, which are used to quantify image quality, while sharpness is related to the definition of the boundaries of an image feature.

**Fig. 9:**
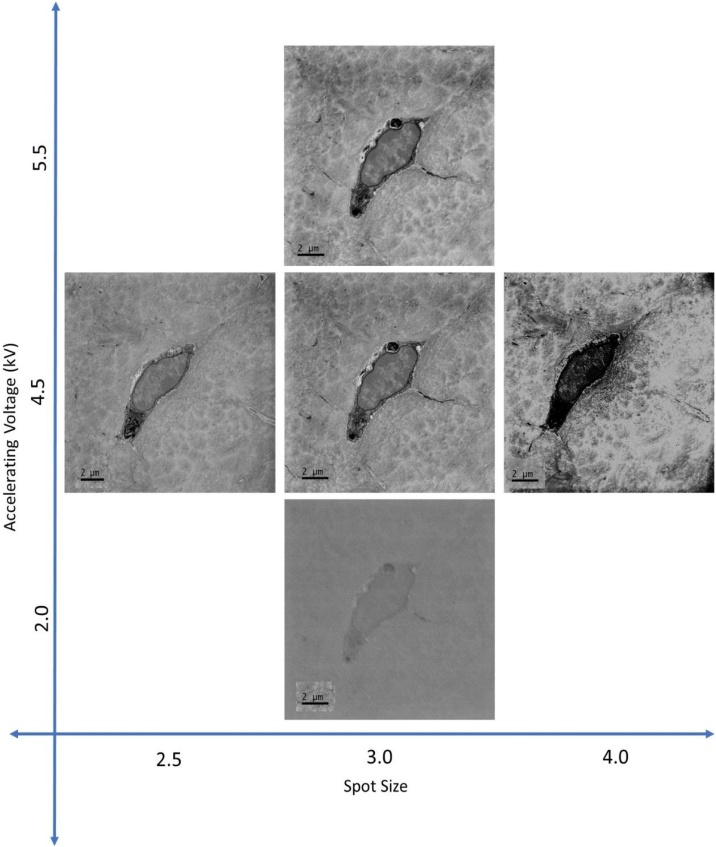
**Effects of varying spot size and accelerating voltage on SBF SEM image quality**. Images were taken using SBF SEM at a pixel size of 3.8 nm, 4k × 4k image size, a dwell time of 4 μs and a chamber pressure of 60.0 Pa. Images captured at lower accelerating voltage showed lower image contrast, while increased accelerating voltage and larger spot size led to damage on the block surface and charging, shown by dark patches in the image and lost detail within the cell. The image on the left shows neither charging nor surface damage and exhibits adequate image contrast to distinguish details of the cell ultrastructure. All scale bars = 2 μm.

A block of decalcified murine bone tissue obtained and prepared for SBF SEM (Section 5 ‘Protocol for SBF SEM imaging of bone tissue’) was imaged at spot size 3, chamber pressure of 60.0 Pa, 4k × 4k image size, 4 nm pixel size and at a dwell time of 4 μs. Keeping these experimental settings fixed, an area of interest containing one osteocyte cell body and surrounding extracellular matrix was imaged while increasing the accelerating voltage in 0.5 kV increments from 2.0 to 5.5 kV (Fig. 9). SEM imaging was repeated on freshly exposed block faces while varying the beam current (spot size 2.5-4.0 at 0.5 intervals), chamber pressure (30–90 Pa at 10 Pa intervals) and slice thickness (100–20 nm at 10 nm intervals). These ranges encompass the extremes of feasible SBF SEM imaging conditions for the used system. To facilitate SNR and CNR calculations, images of blank resin were taken at the same experimental settings. Five rectangular ROIs were defined within each osteocyte nucleus and the standard deviation, minimum, maximum and median grey values were derived using the Analyze > Measure function in the open-source image processing and analysis package Fiji [72]. SNR and CNR were calculated for the ROIs at each experimental setting using the equations below and Matlab (R2016a; The MathWorks, Inc., Natick, MA, USA):

(2) $$SNR=\frac{\tilde{X_{ROI}}}{\sigma_{Blank}}$$

(3) $$CNR=\frac{\left|\tilde{X_{ROI}}-\tilde{X_{Blank}}\right|}{\sigma_{Blank}}$$

with $\tilde{X_{ROI}}$ being the median grey value of the ROI, $\sigma_{Blank}$ the standard deviation of the blank resin image and $\tilde{X_{Blank}}$ the median grey value of the blank resin image. To estimate image sharpness, a ROI was defined at a transition between an osteocyte and the mineralised bone matrix. The edge response assessed in the direction perpendicular to the transition was taken as a measure of image sharpness.

The results are summarised in Table 3. The optimum SNR was observed at an accelerating voltage of 3.5 kV and a spot size of 2.5–3.0, while chamber pressure had no significant effect on the SNR. Optimal CNR was observed at an accelerating voltage of 4.0 kV and a spot size of 3.5, while chamber pressure had no significant effect on the CNR. Sharpness was highest at an accelerating voltage range from 3.0 to 5.5 kV, spot size 3, where lower chamber pressures yielded generally sharper images.

**Table 3:** **Ideal imaging conditions for SBF SEM imaging of bone tissue in the present study.** The imaging conditions were determined by calculating SNR, CNR and sharpness over a range of conditions, selecting the optimum for each and calculating the mean of the results (Section 2.2.3). The practical conditions were determined by visual inspection of images across the same ranges as described in Section 2.2.2.

	**Experimental**	**Practical**
**Accelerating voltage (kV)**	4.5	3.0
**Spot size**	3	3
**Chamber pressure (Pa)**	62.5	60.0
**Dwell time (μs)**	4	4
**Minimum slice thickness possible (nm)**	Not determined	50

### 2.3 Image post-processing and 3D reconstruction

Comprehensive reviews of SBF SEM image processing workflows have been published [32,63]. Software packages and toolkits used with SBF SEM data include both open-source programmes such as Fiji (https://imagej.net/Fiji) [72], Microscopy Image Browser (http://mib.helsinki.fi) [73] and Ilastik (https://www.ilastik.org) [74] as well as commercial software packages such as Avizo (Thermo Fisher Scientific) or Imaris (Oxford Instruments). Often, a combination of these software packages and toolkits is used to process and analyse the 3D datasets.

After collection of datasets several image processing steps must be undertaken before image segmentation and (quantitative) analysis. Using the Gatan 3View® system software DigitalMicrograph®, images are collected and stored as .dm3 or .dm4 files. These files are converted to other file types, typically .tiff or .mrc, which are more widely compatible formats. Binning the image stacks allows the inspection of large volumes and identification of sub-volumes of interest to be extracted. Creating sub-volumes reduces the amount of computational processing power and time required for all subsequent image processing and analysis steps. Image stacks may be filtered, aligned, normalised and the contrast and brightness adjusted if necessary, in order to reduce noise and to support the visual inspection of the images.

**Fig. 10:**
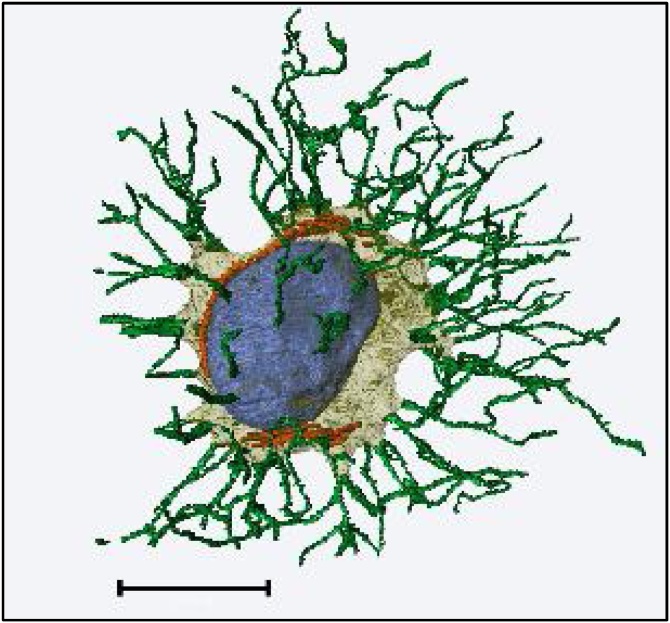
**An osteocyte reconstructed from SBF SEM data of perfusion-fixed, decalcified murine bone, prepared using the sample preparation protocol and imaging conditions described in this publication**. Segmentation and volume rendering were carried out using Avizo. The cell body is shown in pale yellow, processes in green, the nucleus in blue and mitochondria in orange. An interactive .pdf version of this figure is available as a supplementary file (Supplementary 2). Scale bar = 5 μm. (For interpretation of the references to colour in this figure legend, the reader is referred to the web version of this article.)

Segmentation of features of interest can be done either manually (i.e., drawing the outlines of the feature in sequential 2D sections) or in a semi-automated manner, using thresholding, region growing, watershed or machine learning-based classifications. Data visualisation is achieved using 3D volume rendering or surface generation, and outputs such as animations and stereo views can be created. Fig. 10 shows the ultrastructure of a single osteocyte which has been reconstructed from SBF SEM data.

Quantitative measures for segmented features can be derived from datasets using Fiji, Avizo or other software packages and toolkits. When considering the ON&LCN, relevant quantitative measures may include osteocyte volume, osteocyte number density, spatial distribution and alignment, microstructural information including cell, pericellular space and lacunar shape and volume as well as ultrastructural details such as process and canaliculus dimensions and tortuosity.

SBF SEM is a powerful technique with potential to enhance the current state of the art in high-resolution 3D bone imaging. Sample preparation and imaging conditions may both have an impact on image quality variation if care is not taken. It is therefore crucial to consider the following aspects when planning an SBF SEM imaging workflow:

**Table:** 

• **Fixation:** Fixation is the most crucial stage in sample preparation. Inappropriate or delayed fixation can cause shrinkage and allow initiation of autolysis, leading to possible misinterpretation of changes in tissue (ultra)structure. Fixation quality can be checked by collecting a thin tissue section and checking with TEM before proceeding to collect SBF SEM data. • **Staining:** The choice of the adopted tissue staining protocol is important. For thick samples (> 2 mm) specialised staining protocols should be considered to ensure complete and uniform tissue staining. • **Volume of interest:** While the tissue volume that can be imaged using SBF SEM is larger than that using FIB SEM, it is still a relatively small volume compared to what can be assessed by μCT. To avoid sampling error and ensure that a representative tissue volume is imaged, a correlative CT and/or light microscopy approach should be considered, to allow for an informed selection of the (sub-)volume of interest for subsequent SBF SEM imaging [75]. • **Imaging conditions:** Imaging conditions should be carefully chosen and consistently applied. There may be variation in resulting image data from different microscopes. An approach such as detailed in this work (calculating electron dose, calculating SNR, etc.) will ensure the results from different microscopes are comparable. • **Image post-processing and segmentation:** Misinterpretation of images can occur if image post-processing and segmentation workflows are changed during a study. It may be necessary to trial several approaches before selecting and adhering to the most appropriate workflow [76].

## 3 Discussion

SBF SEM is a promising imaging technique which can be applied to hard tissues. SBF SEM facilitates concurrent high-resolution 3D imaging of bone and of osteocytes that are embedded within the bone matrix, including the cell and cell process ultrastructure. SBF SEM can be used to determine whether lacunae and canaliculi are occupied by osteocytes and cell processes, respectively, which is normally not possible when using X-ray-based imaging methods. Care must be taken when using diamond knives to cut calcified material but, operating under appropriate experimental conditions, high-quality images can be obtained. When approaching a new sample, a series of images taken at varying accelerating voltages, spot sizes, chamber pressures and slice thicknesses can be compared using figure of merits characterising image quality, including SNR, CNR and image sharpness as described, representing a useful starting point to determine the ideal imaging conditions for different sample types. The experimental settings for SBF SEM imaging will vary with tissue type, instrumentation, staining protocol and the embedding resin used.

Sample preparation for SBF SEM is important. Immediate tissue fixation is crucial, stopping metabolism and autolytic changes and fixing molecules in their current position. Tissue changes induced by sample preparation, including tissue shrinkage, must be considered during interpretation of the resulting images. The nature of mechanical slicing through microtomy produces typically non-isotropic datasets (different nominal in-plane and out-of-plane resolution), and is inherently destructive to the tissue block. Until automated segmentation routines are improved, image segmentation and processing will remain a bottleneck for quantitative analysis of the resulting images.

Datasets produced by SBF SEM can be investigated qualitatively by visualisation and quantitatively through retrieving morphometric measures, which can be interrogated repeatedly by other investigators. Therefore, in the future, SBF SEM datasets should be placed in open repositories, such as the Electron Microscopy Public Image Archive or EMPIAR (www.ebi.ac.uk/pdbe/emdb/empiar/) for raw data, which allows data sharing and cooperation between researchers. Other initiatives such as the Cell-Centred Database and the Open Microscopy Environment [77,78] also facilitate data sharing and collaboration.

We have demonstrated that SBF SEM is useful technique for imaging both decalcified and undecalcified bone tissue, visualising the ON&LCN simultaneously. The spatial resolution is improved over that achieved with X-ray CT methods and larger volumes of interest may be examined when compared to FIB SEM. The bone tissue preparation and imaging protocols presented here can thus now be applied to address relevant questions in bone research. Quantitative 3D ultrastructural data derived from SBF SEM imaging of osteocytes, including but not limited to the association with the pericellular space and the extracellular matrix, and the distribution of osteocytes within the bone matrix, will add to the understanding of bone mechanobiology and changes in growth, ageing and pathology. SBF SEM imaging of the ON&LCN microstructure and ultrastructure will also contribute to efforts in computational modelling of bone mechanotransduction and mechanosensation.

## 4 Outlook

Developments in BSE detector technology, correlative microscopy [79,75], immunolabelling [80], integration of energy-dispersive X-ray (EDX) systems [81], beam deceleration [82], in-chamber coating [83], developing software capabilities [84], focal gas injection [85] and multibeam SEM imaging [86] will continue to improve the capabilities of SBF SEM imaging. The commercial availability of a suitable conductive resin [67] and methods to increase conductivity of resins [66] will allow improved image quality and a reduction in the amount of heavy metal stains required. Workflows for automated image segmentation are in development [[87], [88], [89]].

Other fields where SBF SEM imaging of hard tissue may be exploited in the future include studies of biomineralised tissue such as enamel, dentine, mineralised mollusc radula, plants, calcified pathological inclusions in tissue, nacre/shell, radiolarians, diatoms, fossilised material and mixed materials such as scaffolds and cells.

## 5 Protocol for SBF SEM imaging of bone tissue

### 5.1 Bone tissue samples

This protocol has been developed for the preparation and SBF SEM imaging of murine and human bone tissue. Collect tissue samples in accordance with the relevant local ethics regulations and legal guidelines. Fixation must be carried out as quickly as possible after excision, to preserve the ultrastructure and to arrest autolytic changes in the osteocytes. Many of the reagents used in this protocol are hazardous. Appropriate risk assessments should be carried out and mitigation measures put in place.

### 5.2 Reagents

**Table:** 

REAGENT	AMOUNT
3% glutaraldehyde (GA) (TAAB Laboratories Equipment Ltd, Aldermaston, UK), 4% formaldehyde (FA) (Fisher Scientific, Loughborough, UK) in 0.1 M piperazinediethane sulfonic acid (PIPES) buffer (Fisher Scientific, Loughborough, UK) pH7.2	
0.1 M PIPES buffer pH7.2	
2% aqueous osmium tetroxide (Oxkem, Reading, UK)	
7% aqueous EDTA (Fisher Scientific, Loughborough, UK)	
Osmium/ferrocyanide (reduced osmium):	
• *Reagents:*	
*3% potassium ferrocyanide (VWR International, Lutterworth, UK) in 0.2 M PIPES buffer (pH7.2)*	*5 ml*
*4% osmium tetroxide*	*5 ml*
• *Method*:	
*Mix the two components together just before use to produce 1.5% potassium ferrocyanide plus 2% osmium tetroxide in 0.1 M PIPES buffer (pH7.2)*	
Distilled water	
Thiocarbohydrazide solution:	
• *Reagents:*	
*Thiocarbohydrazide (Acros Organics, Thermo Fisher Scientific, Geel, Belgium)*	*0.1 g*
*Distilled water*	*10 ml*
• *Method:*	
*Mix the two components together and place in an oven at 60 °C for 1 h (agitate by swirling every 10 min). Filter through 0.22 μm Millipore filter before use.*	
2% aqueous uranyl acetate (Agar Scientific, Stansted, UK)	
Walton’s lead aspartate solution:	
• *Reagents:*	
*Lead nitrate (Agar Scientific, Stansted, UK)*	*0.066 g*
*0.03* *M aspartic acid (Acros Organics, Thermo Fisher Scientific, Geel, Belgium)*	*10 ml*
• *Method:*	
*Mix the two components together and adjust to pH5.5 with 1* *M KOH. Place in oven for 30 min (no precipitate should form).*	
Ethanol series 30%, 50%, 70%, 95%, absolute (Fisher Scientific, Loughborough, UK)	
Acetonitrile (Fisher Scientific, Loughborough, UK)	
Agar low viscosity resin (Spurr replacement) (Agar Scientific, Stansted, UK)	
Toluidine blue (Agar Scientific, Stansted, UK)	
Conductive glue (CircuitWorks Conductive Expoxy CW2400; Chemtronics / ITW Contamination Control Electronics, Kennesaw, GA, USA)	
Contact adhesive/glue (Pattex; Henkel, Düsseldorf, Germany)	

### 5.3 Equipment

**Table:** 

Single-edged razor blades (Fisher Scientific, Loughborough, UK)
Fine-toothed, double-blade ‘razor’ saw (JLC, Czech Republic)
Fine forceps (no preferred supplier)
Glass knives (Agar Scientific, Stansted, UK)
Cocktail sticks or fine paintbrush (no preferred supplier)
Glass vials (Fisher Scientific, Loughborough, UK)
EM grids (EM Resolutions, Sheffield, UK)
Glass slides (no preferred supplier)
Ultramicrotome (no preferred supplier)
Light microscope (no preferred supplier)
Rotator (no preferred supplier)
pH meter (no preferred supplier)
Plastic pipettes (Fisher Scientific, Loughborough, UK)
Oven at 60 °C (no preferred supplier)
Embedding capsules (TAAB Laboratories Equipment Ltd, Aldermaston, UK)
Sputter coater (Quorum Technologies, Laughton, UK)
Aluminium pins (EM Resolutions, Sheffield, UK)

### 5.4 Fixation

It is important that the tissue is never allowed to dry during this protocol. Unless otherwise indicated all stages are carried out at room temperature on a laboratory rotator.

1 Fix tissue by perfusion with 3% GA, 4% FA, in 0.1 M PIPES buffer or if this is not possible, immerse in fixative immediately after excision.
2 Cut blocks of tissue < 2 × 2 × 2 mm^3^ with a single-edged razor blade, diamond saw or fine-toothed, double-blade razor saw from the selected area(s) without allowing the tissue to dry out. Immerse blocks in 3% GA, 4% FA, in 0.1 M PIPES for at least 6 h. Tissue can be stored at 4 °C for up to a week at this point. Rinse in 0.1 M PIPES buffer (2 × 10 min) and place in 2% aqueous osmium tetroxide for 1 h.
3 If samples are to be imaged in the mineralised state, proceed to Section 5.6 Staining.

### 5.5 Decalcification

Decalcify tissue blocks by immersing in 7% EDTA for 1 week, changing the solution daily.

### 5.6 Staining

Immerse tissue in each fluid as indicated below in a glass vial, where ‘RT’ means room temperature. Up to 6 pieces of tissue can be processed in each vial.

**Table:** 

**Solution**	**Temperature**	**Time**
Osmium/ferrocyanide (reduced osmium)	On ice	1 h
Rinse in distilled water	RT	5 x 3 min
Thiocarbohydrazide solution	RT	20 min
Distilled water rinse	RT	5 × 3 min
2% osmium tetroxide	RT	30 min
Distilled water rinse	RT	5 × 3 min
2% uranyl acetate	4 °C	1 h
Distilled water rinse	RT	5 × 3 min
Walton’s lead aspartate solution	60 °C	30 min
Distilled water rinse	RT	5 × 3 min
30% ethanol	RT	10 min
50% ethanol	RT	10 min
70% ethanol	RT	10 min
95% ethanol	RT	10 min
Absolute ethanol	RT	20 min
Absolute ethanol	RT	20 min
Acetonitrile	RT	20 min
50:50 Acetonitrile:ALV resin	RT	Overnight
ALV resin	RT	6 h
Embed in capsules in fresh ALV resin		
ALV resin	60 °C	Overnight

### 5.7 Light microscopy

Semi-thin (0.5-1 μm) sections may be taken, stained with toluidine blue and examined using a light microscope. Ultrathin (∼90 nm) sections may be taken and examined using a TEM before proceeding to SBF SEM block preparation in order to confirm sample orientation, allow selection of an area of interest and confirm tissue fixation quality.

### 5.8 Sample trimming, mounting and SBF SEM imaging

1 Trim the block face with a single-edged razor blade or glass knife to a surface area of < 800 μm^2^ with the region of interest (ROI) near the centre and remove it from the resin block using a sharp, single-edged razor blade. A piece of laboratory film, placed over the surface during removal, can protect against loss of the tissue sub-volume.
2 Attach the block to an aluminium pin using conductive adhesive to enhance conductivity and reduce the build-up of negative surface charge and its adverse effects, such as blurring and distortion of the image.
3 Trim the surface to produce a flat block face using a glass knife or a diamond trimming knife.
4 Sputter-coat the mounted samples with a layer of metal (Au, Pt or Pd) to further reduce the build-up of surface charge.
5 Apply a thin layer of contact adhesive to the edge of the block which will be cut first by the diamond knife.
6 Place the pin in the SBF SEM system.
7 Adjust the height while observing the knife edge and block face. Ensure the ‘stroke-up’ control is activated. This raises the sample by approximately 10 μm for cutting (so that it can drop again when the knife retracts, avoiding contact between the knife and the block).
8 Set the initial settings to 100 nm slice thickness, 100 slices.
9 Start approach and watch until the complete block face is exposed.
10 Move the knife to the original ‘clear’ position and clean debris from the block face and knife edge using an air duster. Close door and pump down chamber.
11 Suggested settings for 3View® 2XP on a FEI Quanta 250 FEGSEM system: accelerating voltage of 3 kV, spot size 3.5, chamber pressure 60.0 Pa, 4k × 4k image size at 5-50 nm pixel size (varies with desired field of view and time available).
12 Optimise focus and stigmator settings.
13 Set slice thickness, number of slices and autofocus.
14 Start data collection.

## 6 Data accessibility

The data supporting this work are openly available from the University of Southampton repository at http://dx.doi.org/10.5258/SOTON/D1089.

## Declaration of competing interests

At the time of this work SS was employed by Gatan UK, which manufactures the 3View® attachment for SBF SEM. He is now co-founder of ConnectomX, which manufactures the Katana Microtome. HG is employed by Diatome, a company manufacturing diamond knives for ultramicrotomy.

## References

[1] Knothe Tate M.L., Adamson J.R., Tami A.E., Bauer T.W. (2004). The osteocyte. Int. J. Biochem. Cell Biol..

[2] Dallas S.L., Bonewald L.F. (2010). Dynamics of the transition from osteoblast to osteocyte. Ann. N. Y. Acad. Sci..

[3] You L., Cowin S.C., Schaffler M.B., Weinbaum S. (2001). A model for strain amplification in the actin cytoskeleton of osteocytes due to fluid drag on pericellular matrix. J. Biomech..

[4] Bonivtch A.R., Bonewald L.F., Nicolella D.P. (2007). Tissue strain amplification at the osteocyte lacuna: a microstructural finite element analysis. J. Biomech..

[5] Goggin P.M., Zygalakis K.C., Oreffo R.O., Schneider P. (2016). High-resolution 3D imaging of osteocytes and computational modelling in mechanobiology: insights on bone development, ageing, health and disease. Eur. Cell. Mater..

[6] Repp F., Kollmannsberger P., Roschger A., Kerschnitzki M., Berzlanovich A., Gruber G.M., Roschger P., Wagermaier W., Weinkamer R. (2017). Spatial heterogeneity in the canalicular density of the osteocyte network in human osteons. Bone Rep..

[7] Verbruggen S.W., Mc Garrigle M.J., Haugh M.G., Voisin M.C., McNamara L.M. (2015). Altered mechanical environment of bone cells in an animal model of short- and long-term osteoporosis. Biophys. J..

[8] Georgiadis M., Müller R., Schneider P. (2016). Techniques to assess bone ultrastructure organization: orientation and arrangement of mineralized collagen fibrils. J. R. Soc. Interface.

[9] Dierolf M., Menzel A., Thibault P., Schneider P., Kewish C.M., Wepf R., Bunk O., Pfeiffer F. (2010). Ptychographic X-ray computed tomography at the nanoscale. Nature.

[10] Andrews J.C., Brennan S., Patty C., Luening K., Pianetta P., Almeida E., van Der Meulen M.C.H., Feser M., Gelb J., Rudati J., Tkachuk A., Yun W.B. (2008). A high resolution, hard x-ray bio-imaging facility at SSRL. Synchrotron Radiat. News.

[11] Holler M., Diaz A., Guizar-Sicairos M., Karvinen P., Färm E., Härkönen E., Ritala M., Menzel A., Raabe J., Bunk O. (2014). X-ray ptychographic computed tomography at 16 nm isotropic 3D resolution. Sci. Rep..

[12] White J.G., Southgate E., Thomson J.N., Brenner S. (1986). The structure of the nervous system of the nematode Caenorhabditis elegans. Philos. Trans. R. Soc. Lond., B, Biol. Sci..

[13] Peddie C.J., Collinson L.M. (2014). Exploring the third dimension: volume electron microscopy comes of age. Micron.

[14] Kamioka H., Honjo T., Takano-Yamamoto T. (2001). A three-dimensional distribution of osteocyte processes revealed by the combination of confocal laser scanning microscopy and differential interference contrast microscopy. Bone.

[15] Verbruggen S.W., Vaughan T.J., McNamara L.M. (2016). Mechanisms of osteocyte stimulation in osteoporosis. J. Mech. Behav. Biomed. Mater..

[16] Vatsa A., Breuls R.G., Semeins C.M., Salmon P.L., Smit T.H., Klein-Nulend J. (2008). Osteocyte morphology in fibula and calvaria – is there a role for mechanosensing?. Bone.

[17] Cooper D.M., Turinsky A.L., Sensen C.W., Hallgrimsson B. (2003). Quantitative 3D analysis of the canal network in cortical bone by micro-computed tomography. Anat. Rec. B New Anat..

[18] Schneider P., Stauber M., Voide R., Stampanoni M., Donahue L.R., Müller R. (2007). Ultrastructural properties in cortical bone vary greatly in two inbred strains of mice as assessed by synchrotron light based micro- and nano-CT. J. Bone Miner. Res..

[19] Langer M., Pacureanu A., Suhonen H., Grimal Q., Cloetens P., Peyrin F. (2012). X-ray phase nanotomography resolves the 3D human bone ultrastructure. PLoS One.

[20] Peyrin F., Salome M., Nuzzo S., Cloetens P., Laval-Jeantet A.M., Baruchel J. (2000). Perspectives in three-dimensional analysis of bone samples using synchrotron radiation microtomography. Cell. Mol. Biol. (Noisy-le-grand).

[21] Suzuki R., Domon T., Wakita M. (2000). Some osteocytes released from their lacunae are embedded again in the bone and not engulfed by osteoclasts during bone remodeling. Anat Embryol (Berl).

[22] Kamioka H., Murshid S.A., Ishihara Y., Kajimura N., Hasegawa T., Ando R., Sugawara Y., Yamashiro T., Takaoka A., Takano-Yamamoto T. (2009). A method for observing silver-stained osteocytes in situ in 3-microm sections using ultra-high voltage electron microscopy tomography. Microsc. Microanal..

[23] Reznikov N., Almany-Magal R., Shahar R., Weiner S. (2013). Three-dimensional imaging of collagen fibril organization in rat circumferential lamellar bone using a dual beam electron microscope reveals ordered and disordered sub-lamellar structures. Bone.

[24] Schneider P., Meier M., Wepf R., Müller R. (2011). Serial FIB/SEM imaging for quantitative 3D assessment of the osteocyte lacuno-canalicular network. Bone.

[25] Tapia J.C., Kasthuri N., Hayworth K.J., Schalek R., Lichtman J.W., Smith S.J., Buchanan J. (2012). High-contrast en bloc staining of neuronal tissue for field emission scanning electron microscopy. Nat. Protoc..

[26] Leighton S.B. (1981). SEM images of block faces, cut by a miniature microtome within the SEM - a technical note. Scan. Electron Microsc..

[27] Leighton S.B., Kuzirian A.M. (1987). Sectionless sectioning - a systematic method for scanning electron microscopic examination of embedded tissue. Biol. Bull..

[28] Denk W., Horstmann H. (2004). Serial block-face scanning electron microscopy to reconstruct three-dimensional tissue nanostructure. PLoS Biol..

[29] Wanner A.A., Kirschmann M.A., Genoud C. (2015). Challenges of microtome-based serial block-face scanning electron microscopy in neuroscience. J. Microsc..

[30] Peretti D., Bastide A., Radford H., Verity N., Molloy C., Martin M.G., Moreno J.A., Steinert J.R., Smith T., Dinsdale D., Willis A.E., Mallucci G.R. (2015). RBM3 mediates structural plasticity and protective effects of cooling in neurodegeneration. Nature.

[31] Eisenstein M. (2009). Neural circuits: putting neurons on the map. Nature.

[32] Borrett S., Hughes L. (2016). Reporting methods for processing and analysis of data from serial block face scanning electron microscopy. J. Microsc..

[33] Tanoue R., Ohta K., Miyazono Y., Iwanaga J., Koba A., Natori T., Iwamoto O., Nakamura K.-I., Kusukawa J. (2018). Three-dimensional ultrastructural analysis of the interface between an implanted demineralised dentin matrix and the surrounding newly formed bone. Sci. Rep..

[35] Hayat M.A. (2000). Principles and techniques of electron microscopy: biological applications.

[36] Mollenhauer H.H. (1993). Artifacts caused by dehydration and epoxy embedding in transmission electron microscopy. Microsc. Res. Tech..

[37] McNamara L.M., Majeska R.J., Weinbaum S., Friedrich V., Schaffler M.B. (2009). Attachment of osteocyte cell processes to the bone matrix. Anat. Rec. (Hoboken).

[38] You L.-D., Weinbaum S., Cowin S.C., Schaffler M.B. (2004). Ultrastructure of the osteocyte process and its pericellular matrix. Anat. Rec. A. Discov. Mol. Cell. Evol. Biol..

[39] Everts V., Niehof A., Tigchelaar-Gutter W., Beertsen W. (2012). Transmission electron microscopy of bone. Methods Mol. Biol..

[40] An Y.H., Martin K. (2003). Handbook of histology methods for bone and cartilage.

[41] Boyde A. (2018). Evaluation of laser ablation microtomy for correlative microscopy of hard tissues. J. Microsc..

[42] Bakhsh T.A. (2016). Ultrastructural features of dentinoenamel junction revealed by focused gallium ion beam milling. J. Microsc..

[43] Bakhsh T.A., Sadr A., Mandurah M.M., Shimada Y., Zakaria O., Tagami J. (2015). In situ characterization of resin-dentin interfaces using conventional vs. cryofocused ion-beam milling. Dent. Mater..

[44] Palamara J., Phakey P.P., Rachinger W.A., Orams H.J. (1981). Electron-microscope study of the dentine-enamel junction of kangaroo (Macropus giganteus) teeth using selected-area argon-ion-beam thinning. Cell Tissue Res..

[45] Page K.M., Stevens A., Lowe J., Bancroft J.D., Bancroft J.D., Stevens A. (1996). Bone. Theory and practice of histological techniques.

[46] Sangeetha R., Uma K., Chandavarkar V. (2013). Comparison of routine decalcification methods with microwave decalcification of bone and teeth. J. Oral Maxillofac. Pathol..

[47] Callis G., Sterchi D. (1998). Decalcification of bone: literature review and practical study of various decalcifying agents, methods, and their effects on bone histology. J. Histotechnol..

[48] Sanjai K., Kumarswamy J., Patil A., Papaiah L., Jayaram S., Krishnan L. (2012). Evaluation and comparison of decalcification agents on the human teeth. J. Oral Maxillofac. Pathol..

[49] Kapila S.N., Natarajan S., Boaz K., Pandya J.A., Yinti S.R. (2015). Driving the mineral out faster: simple modifications of the decalcification technique. J. Clin. Diagn. Res..

[50] Shah F.A., Johansson B.R., Thomsen P., Palmquist A. (2015). Ultrastructural evaluation of shrinkage artefacts induced by fixatives and embedding resins on osteocyte processes and pericellular space dimensions. J. Biomed. Mater. Res. A..

[51] Bonucci E., Reurink J. (1978). The fine structure of decalcified cartilage and bone: a comparison between decalcification procedures performed before and after embedding. Calcif. Tissue Res..

[52] Pitol D.L., Caetano F.H., Lunardi L.O. (2007). Microwave-induced fast decalcification of rat bone for electron microscopic analysis: an ultrastructural and cytochemical study. Braz. Dent. J..

[53] Lin Y., Xu S. (2011). AFM analysis of the lacunar-canalicular network in demineralized compact bone. J. Microsc..

[54] Palade G.E. (1952). A study of fixation for electron microscopy. J. Exp. Med..

[55] Seligman A.M., Wasserkrug H.L., Hanker J.S. (1966). A new staining method (OTO) for enhancing contrast of lipid-containing membranes and droplets in osmium tetroxide-fixed tissue with osmiophilic thiocarbohydrazide (TCH). J. Cell Biol..

[56] Malick L.E., Wilson R.B., Stetson D. (1975). Modified thiocarbohydrazide procedure for scanning electron microscopy: routine use for normal, pathological, or experimental tissues. Stain Technol..

[57] Willingham M.C., Rutherford A.V. (1984). The use of osmium-thiocarbohydrazide-osmium (OTO) and ferrocyanide-reduced osmium methods to enhance membrane contrast and preservation in cultured cells. J. Histochem. Cytochem..

[58] Karnovsky M.J. (1971). Use of ferrocyanide-reduced osmium tetroxide in electron microscopy. 11th Annual meeting of the Americal Society for Cell Biology.

[59] Deerinck T.J., Bushong E.A., Thor A., Ellisman M.H. (2010). NCMIR methods for 3D EM: a new protocol for preparation of biological specimens for serial blockface scanning electron microscopy. https://ncmir.ucsd.edu/sbem-protocol.

[60] Starborg T., Kalson N.S., Lu Y., Mironov A., Cootes T.F., Holmes D.F., Kadler K.E. (2013). Using transmission electron microscopy and 3View to determine collagen fibril size and three-dimensional organization. Nat. Protoc..

[61] Mikula S., Denk W. (2015). High-resolution whole-brain staining for electron microscopic circuit reconstruction. Nat. Methods.

[62] Hua Y., Laserstein P., Helmstaedter M. (2015). Large-volume en-bloc staining for electron microscopy-based connectomics. Nat. Commun..

[63] Kittelmann M., Hawes C., Hughes L. (2016). Serial block face scanning electron microscopy and the reconstruction of plant cell membrane systems. J. Microsc..

[64] Kremer A., Lippens S., Bartunkova S., Asselbergh B., Blanpain C., Fendrych M., Goossens A., Holt M., Janssens S., Krols M., Larsimont J.C., Mc Guire C., Nowack M.K., Saelens X., Schertel A., Schepens B., Slezak M., Timmerman V., Theunis C., Van Brempt R., Visser Y., Guérin C.J. (2015). Developing 3D SEM in a broad biological context. J. Microsc..

[65] Kizilyaprak C., Longo G., Daraspe J., Humbel B.M. (2015). Investigation of resins suitable for the preparation of biological sample for 3-D electron microscopy. J. Struct. Biol..

[66] Nguyen H.B., Thai T.Q., Saitoh S., Wu B., Saitoh Y., Shimo S., Fujitani H., Otobe H., Ohno N. (2016). Conductive resins improve charging and resolution of acquired images in electron microscopic volume imaging. Sci. Rep..

[67] M.H. Ellisman, J.R. Johnson, T.J. Deerinck, E.A. Bushong, J. Bouwer, R. Rumachandra, J.S. Siegel, Highly conductive nanocomposite, biological and small molecule materials for enhanced resin conductivilty. Patent application WO2015009941A1, (2015).

[68] Xipell J.M., Gladwin R.C. (1972). The use of a low-viscosity epoxy resin in the preparation of undecalcified bone sections for light microscopy. J. Microsc..

[69] F. Fernandez-Moran Villalobos, Method of making diamond knives. Patent application US3190047A, (1965).

[70] Hashimoto T., Thompson G.E., Zhou X., Withers P.J. (2016). 3D imaging by serial block face scanning electron microscopy for materials science using ultramicrotomy. Ultramicroscopy.

[71] Kubota Y. (2015). New developments in electron microscopy for serial image acquisition of neuronal profiles. Microscopy (Oxf).

[72] Schindelin J., Arganda-Carreras I., Frise E., Kaynig V., Longair M., Pietzsch T., Preibisch S., Rueden C., Saalfeld S., Schmid B., Tinevez J.Y., White D.J., Hartenstein V., Eliceiri K., Tomancak P., Cardona A. (2012). Fiji: an open-source platform for biological-image analysis. Nat. Methods.

[73] Belevich I., Joensuu M., Kumar D., Vihinen H., Jokitalo E. (2016). Microscopy Image Browser: a platform for segmentation and analysis of multidimensional datasets. PLoS Biol..

[74] Kreshuk A., Straehle C.N., Sommer C., Koethe U., Cantoni M., Knott G., Hamprecht F.A. (2011). Automated detection and segmentation of synaptic contacts in nearly isotropic serial electron microscopy images. PLoS One.

[75] Starborg T., O’Sullivan J.D.B., Carneiro C.M., Behnsen J., Else K.J., Grencis R.K., Withers P.J. (2019). Experimental steering of electron microscopy studies using prior X-ray computed tomography. Ultramicroscopy.

[76] Cocks E., Taggart M., Rind F.C., White K. (2018). A guide to analysis and reconstruction of serial block face scanning electron microscopy data. J. Microsc..

[77] Goldberg I., Allan C., Burel J.-M., Creager D., Falconi A., Hochheiser H., Johnston J., Mellen J., Sorger P.K., Swedlow J.R. (2005). The open microscopy environment (ome) data model and XML file: open tools for informatics and quantitative analysis in biological imaging. Genome Biol..

[78] Martone M.E., Ellisman M.H., Sosinsky G.E., Gupta A., Tran J., Wong W., Cone A.C., Fong L., Maynard S. (2017). Cell centered database.

[79] Brama E., Peddie C.J., Wilkes G., Gu Y., Collinson L.M., Jones M.L. (2016). ultraLM and miniLM: Locator tools for smart tracking of fluorescent cells in correlative light and electron microscopy. Wellcome Open Res..

[80] Vihinen H., Belevich I., Jokitalo E. (2012). Electron tomography and serial block face scanning electron microscopy complement each other in 3D morphological characterization of cell organelles. 15th European Microscopy Congress, 16-21 September, LS2.4.

[81] Zankel A. (2011). 3D elemental mapping in the ESEM- a combination of serial block-face SEM and EDS.

[82] Bouwer J.C., Deerinck T.J., Bushong E., Astakhov V., Ramachandra R., Peltier S.T., Ellisman M.H. (2017). Deceleration of probe beam by stage bias potential improves resolution of serial block-face scanning electron microscopic images. Adv. Struct. Chem. Imaging.

[83] Titze B., Denk W. (2013). Automated in-chamber specimen coating for serial block-face electron microscopy. J. Microsc..

[84] Titze B., Genoud C., Friedrich R.W. (2018). SBEMimage: versatile acquisition control software for serial block-face electron microscopy. Front. Neural Circuits.

[85] Deerinck T.J., Shone T.M., Bushong E.A., Ramachandra R., Peltier S.T., Ellisman M.H. (2018). High-performance serial block-face SEM of nonconductive biological samples enabled by focal gas injection-based charge compensation. J. Microsc..

[86] Tate M.L.K., Zeidler D., Pereira A.F., Hageman D., Garbowski T., Mishra S., Gardner L., Knothe U.R. (2016). Organ-to-cell-scale health assessment using geographical information system approaches with multibeam scanning electron microscopy. Adv. Healthc. Mater..

[87] Perez A.J., Seyedhosseini M., Deerinck T.J., Bushong E.A., Panda S., Tasdizen T., Ellisman M.H. (2014). A workflow for the automatic segmentation of organelles in electron microscopy image stacks. Front. Neuroanat..

[88] Wernitznig S., Sele M., Urschler M., Zankel A., Polt P., Rind F.C., Leitinger G. (2016). Optimizing the 3D-reconstruction technique for serial block-face scanning electron microscopy. J. Neurosci. Methods.

[89] Liu T., Jones C., Seyedhosseini M., Tasdizen T. (2014). A modular hierarchical approach to 3D electron microscopy image segmentation. J. Neurosci. Methods.

