# Supplementary material for: Development of protocols for the first serial block-face scanning electron microscopy (SBF SEM) studies of bone tissue
Source: Bone. 2020 Feb;131:115107. doi: 10.1016/j.bone.2019.115107 (PMC6961117; doi:10.1016/j.bone.2019.115107)
Supplement: Supplementary file 2 [file mmc2.pdf]

Osteocyte Ultrastructure

An osteocyte reconstructed from SBF SEM data of perfusion-fixed, decalcified murine bone prepared using the protocol and image settings described in this paper. Segmentation and volume rendering were carried out using Avizo. The cell body is shown in pale yellow, processes in green, the nucleus in blue and mitochondria in orange.

This is an interactive pdf. Click on the image to activate 3D content. To enable the 3D functions, go to Edit > Preferences > 3D & Multimedia, and check the box “Enable playing of 3D content". The buttons below allow parts of the cell to be viewed separately.

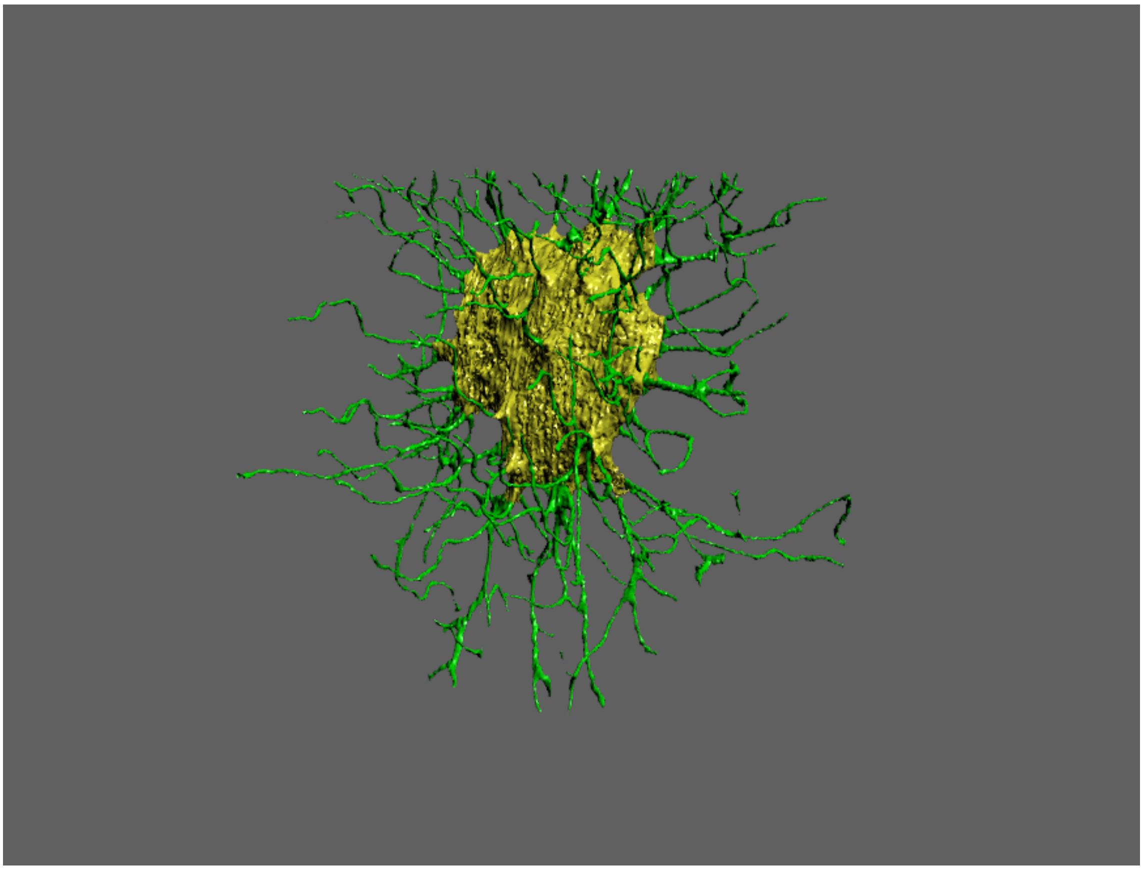

	SHOW	TRANSPARENT	HIDE
Cell body			
Processes			
Nucleus			
Mitochondria			
